# Long Term Outcomes of The Off-Pump and On-Pump Coronary Artery Bypass Grafting In A High-Volume Center

**DOI:** 10.1038/s41598-019-45093-3

**Published:** 2019-06-12

**Authors:** Milos Matkovic, Vladimir Tutus, Ilija Bilbija, Jelena Milin Lazovic, Marko Savic, Marko Cubrilo, Nemanja Aleksic, Igor Atanasijevic, Vuk Andrijasevic, Svetozar Putnik

**Affiliations:** 1 Department for Cardiac surgery, Clinical Center of Serbia, Koste Todorovica 8, 11000 Belgrade, Serbia; 2 Department for Anesthesiology and Intensive care, Clinical Center of Serbia, Koste Todorovica 8, 11000 Belgrade, Serbia; 3 Department for Biostatistics, Faculty of Medicine, University of Belgrade, Doktora Subotica 15, 11000 Belgrade, Serbia; 4 Institute for Cardiovascular diseases Dedinje, Heroja Milana Tepica 1, 11000 Belgrade, Serbia; 5 Faculty of Medicine, University of Belgrade, Doktora Subotica 15, 11000 Belgrade, Serbia

**Keywords:** Cardiology, Acute coronary syndromes

## Abstract

Coronary artery bypass grafting (CABG) remains the most frequent surgery in the practice of an adult cardiac surgeon and the most frequently performed cardiac surgical procedure worldwide. Despite the ongoing debates regarding the superiority or inferiority of off-pump coronary artery bypass grafting, it still comprises 15–30% of all CABG cases varying in different national registries. We performed a propensity matched study of 302 consecutive CABG patients,143 off -pump cases performed by the four experienced off-pump surgeons and the on-pump CABG cases performed by those surgeons and four other experienced coronary surgeons. The five year follow up was performed and data collected comprised of mortality, rehospitalization due to cardiac origin, repeated revascularization, myocardial infarction and cerebrovascular accident. Overall, the off-pump group of patients had a higher risk profile than the patients in the on-pump group. After matching, fewer differences were found between the groups. Propensity score matching analysis showed no difference in long-term survival as well as MACCE and repeated revascularization. The higher risk profile of the patients subjected to OPCAB and the comparable survival to lower risk CPB patients in this series indicate that in experienced hands, OPCAB is a valuable option in this important subgroup of patients.

## Introduction

Coronary artery bypass grafting (CABG) laid the foundation for the modern era of cardiac surgery and remains the most frequent surgery in the practice of an adult cardiac surgeon. Despite all ongoing debates regarding off-pump and on-pump CABG, we cannot ignore the fact that the first CABG interventions were performed on beating hearts^1^. The interest for the off-pump coronary surgery (OPCAB) option arose in the 90’s and led to creation of numerous papers managing this subject spanning from superiority to non-inferiority of OPCAB.

The largest randomized study comparing OPCAB with conventional surgery (the CORONARY trial) clearly showed no significant difference in terms of survival after five year follow up^2^. Several broad systematic reviews and meta-analyses questioned the superiority of OPCAB over conventional surgery with some of them showing better survival for the latter^3–5^. However, all of them have shown that results were biased by underexperienced off-pump surgeons (<20 cases in the ROOBY trial) as well as low volume centers, with some of them having less than two OPCAB cases per month. As stated by Kirmani *et al*., the lack of experience was an important issue in several published national registries and propensity score matching studies^6^.

Despite the ongoing debates regarding the superiority or inferiority of OPCAB, it still comprises 15–30% of all CABG cases varying in different national registries.

Thus, we decided to compare long-term results of OPCAB surgery vs on-pump surgery in “real-world” framework – in a high-volume center with experienced OPCAB surgeons.

## Methods

### Design

A retrospective, observational cohort study with prospectively collected data and propensity score matching was performed.

### Patients

We included 302 patients subjected to isolated CABG, either off- or on-pump during 2012–2013 performed by experienced surgeons in the department for cardiac surgery. The 143 off-pump cases were consecutive patients operated on by four surgeons with more than 250 previous OPCAB cases per surgeon. The control group comprised of 159 patients operated on-pump during the same period of time by the aforementioned four surgeons and another four experienced on-pump surgeons who performed >95% of their surgeries on-pump. The OPCAB surgeon group had a mixed practice with 30–35% of their CABG practice consisting of OPCAB cases. All surgeons who participated in the study had already performed more than 700 CABG cases and more than 1000 cardiac surgery procedures. CABG surgeries performed by less experienced surgeons or trainee first operators were excluded from the study. The share of OPCAB surgeries in our department is about 25% of all CABG surgeries performed every year. The choice of OPCAB or on-pump surgery is left at surgeons’ discretion. The study was designed as intention-to-treat.

The ethical board of our institution approved the study protocol and data gathering and waived for a need of informed consent form patients for data usage. All patients signed the informed consent on follow up.

### Data collection

The preoperative patient characteristics, operative data and in-hospital mortality and complications were prospectively entered in a hospital board approved database. The follow up data comprised of mortality, rehospitalization due to cardiac origin, repeated revascularization, myocardial infarction and cerebrovascular accident. Repeated revascularization was defined as either PCI intervention or repeated surgery. The definitions of all variables were taken from either STS score or Euroscore II variable definitions. The follow-up data were gathered by telephone interviews or from the scheduled follow-up examination.

### Surgical technique

All surgeries were performed under general endotracheal anesthesia. OPCAB procedures were done with the aid of deep pericardial stiches and heart stabilizers for the exposure of coronary arteries. The Octopus device and the Starfish device stabilizers were used in all cases (Medtronic Inc., Minneapolis, MN). Intracoronary shunts were used for myocardial protection in all cases and silastic coronary slings were used to facilitate their placement.

The on-pump cases were performed under standard cardiopulmonary bypass (CPB) procedure with ascending aortic cannulation and cannulation of the right atrium with a single two-staged cannula. Anterograde crystalloid St.Thomas’ cardioplegic solution was used to arrest the heart.

The grafts used were left and right internal mammary artery either *in situ* or on “Y” construction as well as saphenous vein grafts.

### Statistical analysis

Descriptive statistics was calculated for the baseline demographic and clinical features, as well as treatment outcomes. Normality of distribution was tested by graphical and mathematical methods. Continuous variables were presented as means with standard deviations or median with 25^th^–75^th^ percentile as appropriate. Categorical variables were presented as numbers and percentages. Differences between groups were analyzed using Students t-test for continuous variables (or Mann Whitney test) and the Pearson chi-squared test for categorical variables.

Survival was computed by use of the Kaplan-Meier survival analysis, and the 2 groups were compared using a log-rank test. Propensity matching of OFF and ON group was performed 1:1 for the extent of disease with greedy, nearest neighbor matching without replacement and a caliper of 0.2. The variables used for propensity matching calculations were age, gender, chronic kidney disease, diabetes and ejection fraction. To maximize inclusion of patients, the Cox regression analysis adjusted for propensity was performed on both matched and unmatched patients. The full code for the analysis is available in Data Supplement Appendix I. The level of significance was set at 0.05. Statistical analysis was performed using the IBM SPSS 21 (Chicago, IL, 2012) package.

## Results

**Figure 1:**
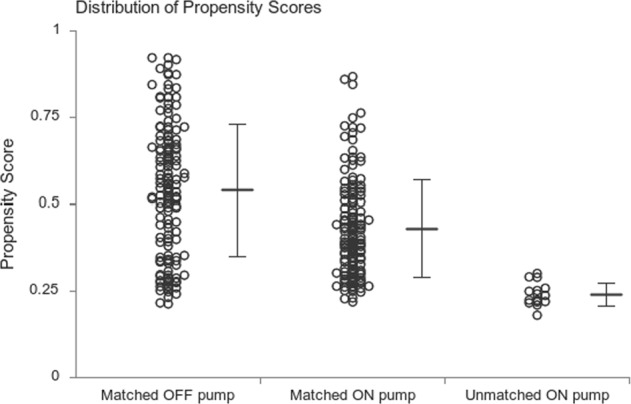
Dot plot of propensity scores of matched and unmatched patients.

The preoperative patient characteristics of the unmatched and matched groups are presented in Table 1. Propensity matching of these two groups was performed 1:1 for the extent of the disease with greedy matching for age, gender, chronic kidney disease, diabetes and left ventricular ejection fraction (LVEF). The distribution of propensity scores is given in Fig. 1. Patients in the OPCAB group had a higher proportion of diabetes mellitus and chronic kidney disease. There was a statistically significant difference in 1, 2 and 3 vessel diseases between groups, with higher percentage of one vessel disease observed in the OPCAB group. Also, lower LVEF was registered within the patients in this group. Higher Euroscore II and the STS score was observed in the OPCAB group. Overall, this group of patients had a higher risk profile than the patients in the on-pump group. After matching, fewer differences were found between the groups.

**Table 1:** Preoperative patient characteristics.

variable	measure	Unmatched			Matched		
		0FF pump N = 143	ON N = 159	p	0FF N = 143	ON N = 143	p
Sex	n (%)			0.830			1.000
male		105 (73.4)	115 (72.3)		105 (73.4)	105 (73.4)	
female		38 (26.6)	44 (27.2)		38 (26.6)	38 (26.6)	
Age	mean ± sd	63.8 ± 8.7	63.4 ± 8.2	0.684	63.8 ± 8.7	63.8 ± 8.3	1.000
BMI	mean ± sd	27.3 ± 4.2	27.1 ± 4.0	0.540	27.3 ± 4.2	27.1 ± 4.0	0.613
HGB	mean ± sd	127.8 ± 16.4	129.9 ± 16.4	0.264	127.8 ± 16.4	130.6 ± 16.5	0.153
HTA	n (%)	138 (96.5)	153 (96.2)	0.898	138 (96.5)	139 (97.2)	0.735
HLP	n (%)	111 (77.6)	126 (79.2)	0.732	111 (77.6)	114 (79.7)	0.665
DM	n (%)	56 (39.2)	43 (27.0)	0.025	56 (39.2)	43 (30.1)	0.106
CRF	n (%)	28 (19.6)	9 (5.7)	<0.001	28 (19.6)	9 (6.3)	0.001
COPD	n (%)	7 (4.9)	11 (6.9)	0.458	7 (4.9)	10 (7.0)	0.453
PVD	n (%)	12 (8.4)	10 (6.3)	0.483	12 (8.4)	9 (6.3)	0.496
IM	n (%)	81 (56.6)	97 (61.0)	0.442	81 (56.6)	90 (62.9)	0.278
PCI	n (%)	76 (53.1)	72 (45.3)	0.172	76 (53.1)	66 (46.2)	0.237
ATRIAL FIBRILATION	n (%)	77 (53.8)	76 (47.8)	0.294	77 (53.8)	70 (49.0)	0.408
CVA	n (%)	10 (7.0)	8 (5.0)	0.472	10 (7.0)	7 (4.9)	0.453
Number of grafts				0.077			0.051
1		20 (14.0)	10 (6.3)		20 (14.0)	8 (5.6)	
2		22 (15.4)	24 (15.1)		22 (15.4)	21 (14.7)	
3		101 (70.6)	125 (78.6)		101 (70.6)	114 (79.7)	
Ejection fraction	mean ± sd	45.5 ± 14.3	53.5 ± 10.8	<0.001	45.5 ± 14.3	52.1 ± 10.4	<0.001
EF	n (%)			<0.001			<0.001
low		58 (40.6)	14 (8.8)		58 (40.6)	14 (9.8)	
normal		85 (59.4)	145 (91.2)		85 (59.4)	129 (90.2)	
Euroscore	mean ± sd	3.0 ± 2.7	1.9 ± 3.2	<0.001	3.0 ± 2.7	2.0 ± 3.3	<0.001
STS score	mean ± sd	7.7 ± 5.6	3.2 ± 4.9	<0.001	7.7 ± 5.6	3.3 ± 5.1	<0.001

BMI, body mass index; CRF, chronic renal failure; COPD, chronic obstructive pulmonary disease; CVA, cerebrovascular accident; MI, myocardial infarction; PCI, percutaneous coronary intervention medn, median QRT, quartiles; SD, standard deviation.

Postoperative outcomes were similar between unmatched and matched patients (Table 2). Duration of surgery, ICU stay and total hospital stay were longer in the on-pump group. Median ICU stay was 2 and 3 days for the off- and on-pump group respectively and total hospital stay was 7 days for the off-pump and 10 days for the on-pump group, both variables reaching statistical significance. Fewer patients required blood transfusion postoperatively in the OPCAB group (39,2% vs 62,3%, p < 0,05). The complication rate, comprising of total pulmonary, gastrointestinal and neurological complications did not reach a statistical significance between the groups.

**Table 2:** Postoperative Outcomes.

variable	measure	Unmatched			Matched		
		0FF pump N = 143	ON 1 N = 159	p	0FF N = 143	ON 1 N = 143	p
Surgery duration	medn (QRT)	3.5 (3.0–4.0)	5.0 (4.0–5.0)	<0.001	3.5 (3.0–4.0)	5.0 (4.0–5.0)	<0.001
ICU	medn (QRT)	2.0 (2.0–3.0)	3.0 (3.0–5.0)	<0.001	2.0 (2.0–3.0)	3.0 (3.0–5.0)	<0.001
Hospitalization	medn (QRT)	7.0 (6.0–8.0)	10.0 (7.0–13.0)	<0.001	7.0 (6.0–8.0)	10.0 (7.0–13.0)	<0.001
Transfusion	n (%)	56 (39.2)	99 (62.3)	<0.001	56 (39.2)	85 (59.4)	<0.001
Mortality follow up	n (%)	29 (20.3)	22 (13.8)	0.136	29 (20.3)	19.0 (13.3)	0.114
CVA follow up	n (%)	2 (1.4)	4 (2.5)	0.487	2 (1.4)	4 (2.8)	0.684
MI follow up	n (%)	3 (2.1)	3 (1.9)	0.896	3 (2.1)	3 (2.1)	1.000
Repeated Revascularization	n (%)	5 (3.5)	5 (3.1)	0.865	5 (3.5)	5 (3.5)	1.000
Complications		33 (23.1)	38 (23.9)	0.866	33 (23.1)	33 (23.1)	1.000
Rehospitalization	n (%)	30 (21.0)	19 (11.9)	0.034	30 (21.0)	16 (11.2)	0.024

CVA, cerebrovascular accident; MI, myocardial infarction; PCI, percutaneous coronary intervention medn, median QRT, quartiles; SD, standard deviation.

The median follow-up was 44 months (1–72 months). At the long-term follow- up the two groups did not statistically differ neither in terms of myocardial infarction or cerebrovascular accident, nor in terms of repeated revascularization. However, patients in the OPCAB group had a statistically significant higher rate of repeated hospitalization due to cardiac origin – 21%, comparing to the 11% in the on-pump group. The results persisted after propensity score matching. This could be explained by the choice of the off-pump technique for much higher risk patients by the surgeons which is confirmed by persistence of the difference in LV function and renal failure after propensity matching. The rate of conversion to on-pump was 1,1%, with a reduced long-term survival for these patients.

**Figure 2:**
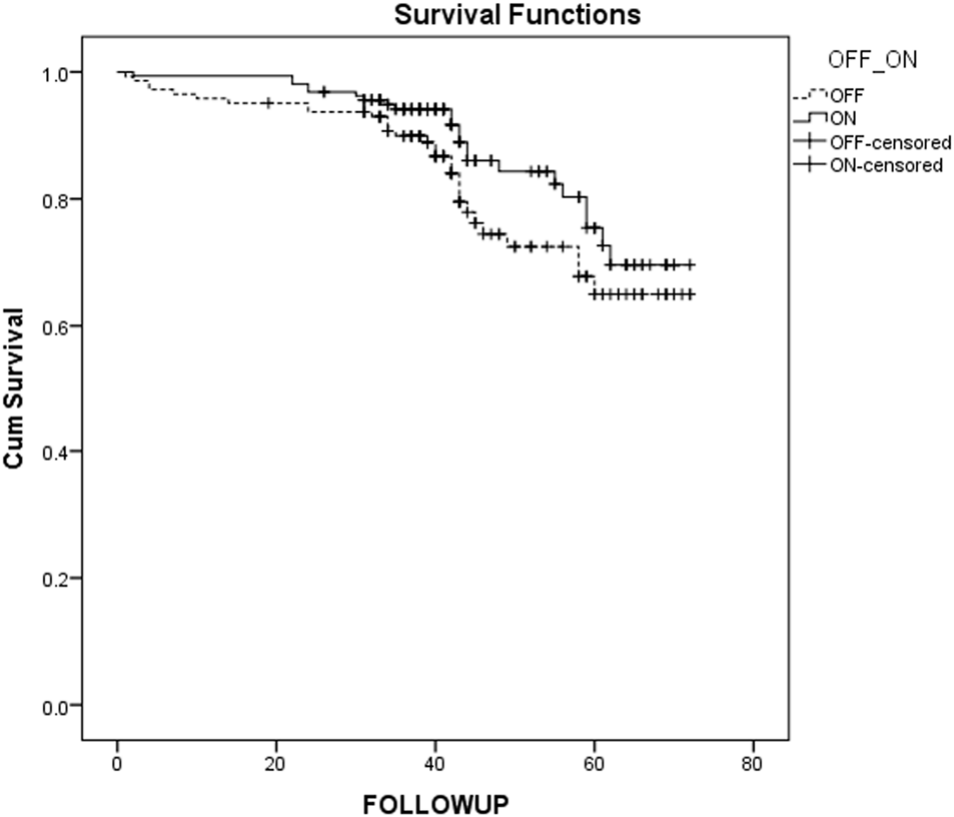
Kaplan-Meier survival curves for all patients.

**Figure 3:**
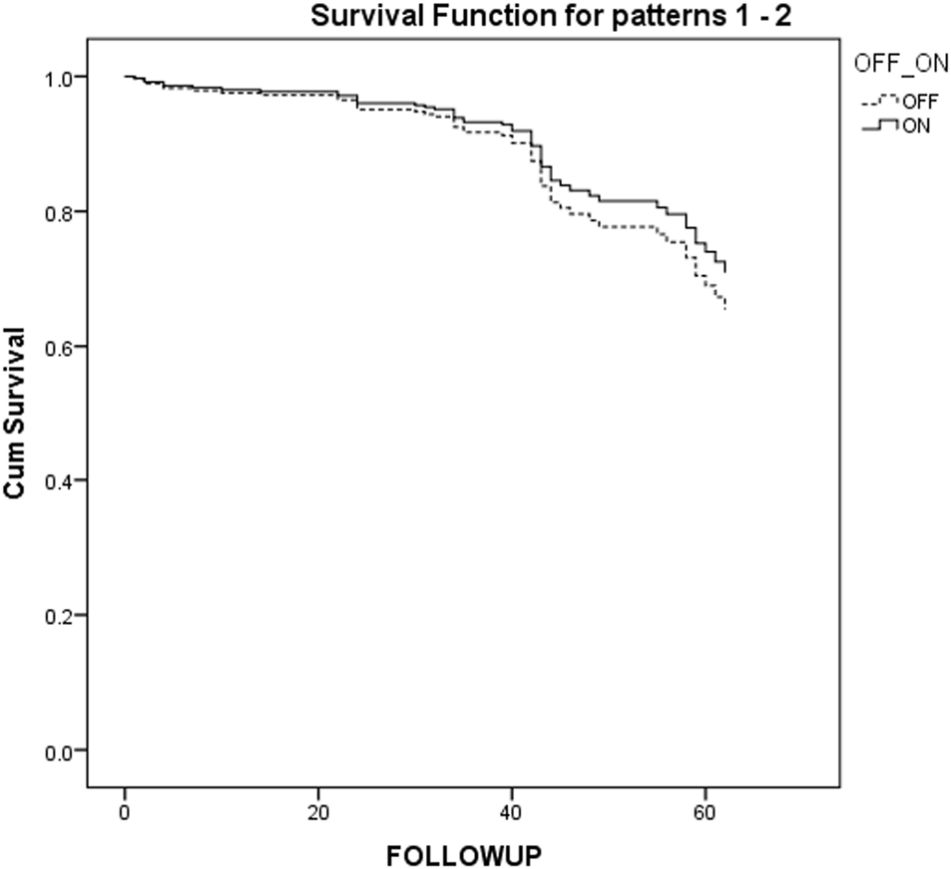
Cox regression adjusted for propensity score.

In unadjusted analysis which included all patients, the mean survival was 60.6 (95% CI 57.094-64.282) months in the off-pump group and 64.5 months (95% CI 61.828–67.362) in the on-pump group. There was no statistically significant difference in long-term survival in unmatched patients over follow-up (Fig. 2, log-rank *P* = 0.1132). In an analysis adjusted for propensity score matching, results persisted – there was no statistically significant difference between on-pump and off-pump groups. The adjusted hazard ratio (HR) of long-term survival was 1.242 (95% confidence interval [CI]: 0.676–2.281; *P* = 0.485 for on- vs off-pump, Fig. 3). No statistical difference regarding mortality was found by surgeons participating in the study.

## Discussion

Our results demonstrate that OPCAB surgery is not inferior to on-pump CABG surgery. Moreover, these results were still obtained even though patients exposed to OPCAB surgery had a higher-risk profile and a lower LVEF compared to patients treated with on-pump CABG surgery.

The interest in OPCAB surgery arose because surgeons saw the opportunity to avoid the adverse events of CPB^7^. The initial clinical use triggered the researchers’ interest in superiority in terms of survival over CPB CABG. One of the first large RCT, the ROOBY trial, showed better survival in the CPB group, as well as better graft patency^8^. Followed by several meta-analyses and reviews with the same result, the questions of surgeon experience and the lengthy follow up period were raised^3,4^. In the ROOBY trial alone, surgeons performed less than 20 previous OPCAB cases before the study and a high conversion rate of 12,4%. Also, the number of OPCAB cases in the meta-analyses frequently shows a number of less than five cases per month per department. In our study, all OPCAB group surgeons have previously performed more than 250 off-pump cases, and all the surgeons included in the study have previously performed more than 700 CABG cases and more than 1000 adult cardiac surgeries. The OPCAB surgeons had a mixed practice with the share of 30–35% of their CABG cases performed off-pump. The number of OPCAB cases in our department is up to 25% of all CABG cases annually, and the OPCAB program began 23 years ago.

The largest RCT with long term follow up, CORONARY follow up, showed no significant difference in survival after 1, 3 and 5 years. Our results from the median follow up of 44 months (ranging 1–72 months) were in line with results from the CORONARY trial, with no significant difference persisting after propensity score matching. The rate of postoperative complications comprising of pulmonary, gastrointestinal or neurological adverse events was not statistically significant, which has already been confirmed in the CORONARY trial. Carmona *et al*. performed a propensity score matching analysis for over 2000 patients over 20 years and found no significant difference in survival, but found lower incidence of postoperative pulmonary, neurological and renal complications in the OPCAB group. All surgeons participating in the study had more than 10 years of experience and had a dedicated OPCAB training^9^. Kirmani *et al*. performed the largest single institution propensity score matching study with over 5000 patients in each group^6^. They also found no statistically significant difference in survival after long-term follow up. They have reported that about 45% of their CABG practice was performed off-pump, with bimodal distribution of surgeons performing their CABG cases >90% whether off- or on-pump. However, their results show a higher incidence of postoperative myocardial infarction within the CPB group. Both studies confirmed shorter ICU and hospital stay, as well as reduced need for blood transfusion for the OPCAB patients, which was also confirmed in our results, persisting after propensity score matching. These findings have been also supported in the large contemporary meta-analysis by Dieberg *et al*.^10^.

The benefits of OPCAB were demonstrated in high risk patients with advanced age, severe calcified aortic disease, impaired left ventricular function and chronic kidney disease^11–13^. Lemma *et al*. conducted a RCT and showed a lower incidence of composite endpoint (operative mortality, myocardial, renal and pulmonary complications) in the high-risk patients subjected to off-pump CABG^14^. Puskas *et al*. supported these results, showing lower operative mortality in high-risk patients and the rise of the benefits with the rise of the operative risk^15^. In our study, patients in the OPCAB had a higher rate of chronic kidney failure, diabetes and impaired ejection fraction. The higher risk profile within the OPCAB group (Euroscore 2,9 ± 0,26 vs 1,9 ± 0,31, STS score 7,7 ± 0,53 vs 3,2 ± 0,49 respectively) showed that surgeons were prone to subject the patients with higher operative risk to the OPCAB procedure. The higher repeated rehospitalization rate due to cardiac origin in the OPCAB group supports the evidence that higher risk patients are more often treated with OPCAB technique. Survival analysis showed no significant difference comparing to lower risk CPB patient profile and the result persisted after propensity score matching. The current ESC/EACTS guidelines suggest that high risk profile patients should be subjected to OPCAB at a high volume experienced off-pump center with a class of evidence IIa^16^.

Since the first studies on this issue emerged, one of the main drawbacks of OPCAB was incomplete revascularization and graft patency. Khan *et al*. performed a RCT showing lower graft patency after three months in the OPCAB group^17^. These findings were supported by Deppe *et al*. in his meta-analysis^5^. However, Puskas *et al*. challenged those findings in their long-term follow up RCT where they showed similar graft patency and completeness of revascularization to CPB after mean follow up of 7 years, stating that the limiting factor of other studies was surgeon experience and center volume as well as high rate of conversion to CPB. Also, the equal patency was demonstrated in the latest analysis by Saki *et al*. in a 3 year follow up period^18^. Calafiore *et al*. state that many of these series had a conversion rate of 10%, with a mortality of conversion up to 16,5%^19^. The conversion rate in our study was 1,1% and the mean number of grafts did not differ between the groups. However, the patency rate was not assessed; the repeated revascularization was used as a clinical correlation parameter and was not statistically different after long-term follow up. The average number of grafts did not differ between the groups, with the exception of one vessel disease, for which the surgeons were more prone to perform on a beating heart.

Our study has several limitations. First, alongside the propensity score matching design and the observational type of study, we were limited by the number of patients. Second, the decision on the type of CABG was left to the surgeon’s preference. Finally, the graft patency was not examined angiographically, instead, the rate of repeated revascularization was used as a clinical correlation parameter.

## Conclusion

In a single experienced OPCAB center propensity score matching analysis there was no difference in long-term survival as well as MACCE and repeated revascularization. The higher risk profile of the patients subjected to OPCAB and the comparable survival to lower risk CPB patients in this series indicate that in experienced hands, OPCAB is a valuable option in this important subgroup of patients. Further, broader studies are needed to investigate whether off-pump surgery should be the standard in experienced and specialized/high-volume centers.

## Supplementary information

Propensity match analysis code

## Data Availability

The datasets used and/or analyzed during the current study available from the corresponding author on reasonable request.
