# Supplementary material for: Long Term Outcomes of The Off-Pump and On-Pump Coronary Artery Bypass Grafting In A High-Volume Center
Source: Sci Rep. 2019 Jun 12;9:8567. doi: 10.1038/s41598-019-45093-3 (PMC6561934; doi:10.1038/s41598-019-45093-3)
Supplement: Supplementary file 1 — Propensity match analysis code [file 41598_2019_45093_MOESM1_ESM.docx]

LONG TERM OUTCOMES OF THE OFF-PUMP AND ON-PUMP CORONARY ARTERY BYPASS GRAFTING IN A HIGH-VOLUME CENTER

Milos Matkovic ^1*^, Vladimir Tutus ^2^, Ilija Bilbija^1,5^, Jelena Milin Lazovic^3^, Marko Savic^3^, Marko Cubrilo^1^, Nemanja Aleksic^1^, Igor Atanasijevic ^4^, Vuk Andrijasevic ^1^ and Svetozar Putnik^1,5^

^1^Department for Cardiac surgery, Clinical Center of Serbia, Koste Todorovica 8, 11000, Belgrade, Serbia

^2^ Department for Anesthesiology and Intensive care, Clinical Center of Serbia, Koste Todorovica 8, 11000, Belgrade, Serbia

^3^Department for Biostatistics, Faculty of Medicine, University of Belgrade, Doktora Subotica 15, 11000, Belgrade, Serbia

^4^ Institute for Cardiovascular diseases Dedinje, Heroja Milana Tepica 1, 11000, Belgrade, Serbia

^5^ Faculty of Medicine, University of Belgrade, Doktora Subotica 15, 11000, Belgrade, Serbia

* Correspondence and requests for materials should be addressed to M.M.

(email: dr.matko@hotmail.com)

Data Supplement Appendix I.

library(readxl)

library(MatchIt)

library(dplyr)

setwd("~/offpump")

dataOR <- read_xlsx("offpump.xlsx")

# Get only variables needed for propensity score matching

# Other variables may have missing values so we don't need to do unnecessary imputation

dataSKR <- data.frame(dataOR$rbr, dataOR$pol, dataOR$god, dataOR$DM, dataOR$HBI, dataOR$EF, dataOR$OFF_ON)

colnames(dataSKR) <- c("rbr","pol","god","DM","HBI","EF","OFF_ON")

# Propensity Matching -----------------------------------------------------

match1 <- matchit(OFF_ON~god+DM+HBI+EF, data=dataSKR, exact=c("pol"), method="nearest", caliper=0.2)

match1.data <- match.data(match1)

# Pooled PSM --------------------------------------------------------------

PSM.pool.1 <- dataSKR

PSM.pool.1$PScore <- match1$distance

PSM.poolmatch.1 <- matchit(OFF_ON~god+DM+HBI+EF, data=PSM.pool.1, exact=c("pol"), method="nearest", distance=PSM.pool.1$PScore)

PSM.matchonly.1 <- match.data (PSM.poolmatch.1)

PSM.matchonly.1$matched <- TRUE

PSM.result <- full_join(PSM.pool.1,PSM.matchonly.1)

PSM.result$matched[is.na(PSM.result$matched)]<- FALSE

dataOUT <- dataOR

dataOUT$PScore <- PSM.result$PScore

dataOUT$matched <- PSM.result$matched

write.csv(dataOUT,file="output.csv",na="")
